# A Movement in the Right Direction

**Published:** 1873-02

**Authors:** 


### *A Movement in the Right Direction.*

We notice that the proprietors of the well-known Crystal Springs (Yates Co., N. Y.), instead of prostituting the curative value of their mineral springs by establishing a "water cure," under the management of some blatant *pathist*, with a wider and more rational view of the real relations of mineral waters to therapeutics, have secured the advice and attendance of a physician of acknowledged ability, professional learning and experience, W. R. Marsh, M.D.; for many years occupying a chair in the Iowa Medical College at Keokuk, and during the whole of the late war serving as regimental and staff surgeon in the U. S. Army. The state of Dr. Marsh's health having obliged him to retire, at least temporarily, from general practice in this city, where he has been located since the close of the war, he has entered upon the duties of this position, determined that there shall be at least one resort for invalids, where physicians may send them without fear of blundering incapacity or bald quackery in treatment.

We cordially recommend the example set by the proprietors of the Crystal Springs to similar establishments throughout the country. Let us have these powerful adjuvants to the treatment of disease, everywhere put under enlightened control.

